# Association of 5-FU Therapeutic Drug Monitoring to DPD Phenotype Assessment May Reduce 5-FU Under-Exposure

**DOI:** 10.3390/ph13110416

**Published:** 2020-11-23

**Authors:** Marine Dolat, Pauline Macaire, Françoise Goirand, Julie Vincent, Audrey Hennequin, Rémi Palmier, Leïla Bengrine-Lefevre, François Ghiringhelli, Bernard Royer, Antonin Schmitt

**Affiliations:** 1 Centre Georges-François Leclerc, 21000 Dijon, France; marine.dolat@hotmail.fr (M.D.); pmacaire@cgfl.fr (P.M.); jvincent@cgfl.fr (J.V.); ahennequin@cgfl.fr (A.H.); rpalmier@cgfl.fr (R.P.); lbengrine@cgfl.fr (L.B.-L.); fghiringhelli@cgfl.fr (F.G.); 2 INSERM U1231, University of Burgundy Franche-Comté, 21000 Dijon, France; 3 Laboratoire de Pharmacologie/Toxicologie, CHU de Dijon, 21000 Dijon, France; francoise.goirand@chu-dijon.fr; 4 Laboratoire de Pharmacologie Clinique, CHU Jean-Minjoz, 3, Boulevard Alexandre-Fleming, 25030 Besançon, France; broyer@chu-besancon.fr; 5 INSERM, EFS BFC, UMR1098, Interactions Greffon-Hôte-Tumeur/Ingénierie Cellulaire et Génique, Université Bourgogne Franche-Comté, 25000 Besançon, France

**Keywords:** DPD, therapeutic drug monitoring, uracilemia, UH_2_/U ratio, GI cancer, 5-FU, pharmacokinetics

## Abstract

In order to limit 5-fluorouracil (5-FU) toxicity, some health agencies recommend evaluating dihydropyrimidine dehydrogenase (DPD) deficiency before any 5-FU treatment introduction. In our study, we investigated relationships between 5-FU clearance and markers of DPD activity such as uracilemia (U), dihydrouracilemia (UH_2_)/U ratio, or genotype of the gene encoding DPD (*DPYD*). All patients with gastrointestinal cancers who received 5-FU-based regimens form March 2018 to June 2020 were included in our study. They routinely benefited of a pre-therapeutic *DPYD* genotyping and phenotyping. During 5-FU infusion, blood samples were collected to measure 5-FU steady-state concentration in order to adapt 5-FU doses at the following cycles. A total of 169 patients were included. Median age was 68 (40–88) years and main primary tumor sites were colorectal (40.8%) and pancreas (31.4%), metastatic in 76.3%. 5-FU was given as part of FOLFIRINOX (44.4%), simplified FOLFOX-6 (26.6%), or docetaxel/FOLFOX-4 (10.6%). Regarding DPD activity, median U and UH_2_/U were, respectively, 10.8 ng/mL and 10.1, and almost 15% harbored a heterozygous mutation. On the range of measured U and UH_2_/U, no correlation was observed with 5-FU clearance. Moreover, in patients with U < 16 ng/mL, 5-FU exposure was higher than in other patients, and most of them benefited of dose increase following 5-FU therapeutic drug monitoring (TDM). If recent guidelines recommend decreasing 5-FU dose in patients harboring U ≥ 16 ng/mL, our study highlights that those patients are at risk of under-exposure and that 5-FU TDM should be conducted in order to avoid loss of efficacy.

## 1. Introduction

5-Fluorouracil (5-FU) is widely used in the treatment of solid malignancies, being the backbone cytotoxic prescribed in gastrointestinal cancers [1]. Despite some advances in its management, 10–30% of patients treated with fluoropyrimidines as a monotherapy experience severe treatment-related toxicity leading to death in 0.5–1% of patients [2,3,4], with this number being even higher when dosed with irinotecan and/or oxaliplatin [5]. Consequently, determination of biomarkers that could predict toxicity of chemotherapeutic agents, including 5-FU, remains a central goal of recent research in oncology.

The most well-known cause of 5-FU intolerance is deficiency of dihydropyrimidine dehydrogenase (DPD) activity—the key enzyme responsible for its metabolism [6]. Complete DPD deficiency is somehow rare and observed in only 0.1 to 0.5% of the population, whereas partial DPD deficiency is observed in 3 to 15% of the population [7,8,9]. Additionally, DPD deficiency is observed in 39–61% of patients developing severe toxicity [3]. As a result, polymorphisms in the gene encoding DPD (*DPYD*), the gene encoding DPD, have gained widespread attention as predictors of fluoropyrimidine-related toxicity. More than 30 sequence variations in the *DPYD* gene have been yet identified, while the most well-established variant is DPYD*2A [10]. The Clinical Pharmacogenetics Implementation Consortium has established the fluoropyrimidines dosage algorithm on the basis of the interpretation of clinical *DPYD* genotype tests. Initial dose reduction of at least 50% is proposed in patients heterozygous for DPYD*2A, DPYD*13, and c.2846A>T who are considered to have intermediate or partial DPD enzyme activity, while a choice of alternative drug is strongly recommended for patients with complete DPD deficiency [11]. However, DPD activity is regulated not only at the level of *DPYD* gene, but also at the transcriptional and the post-transcriptional levels [4]. This highlights the significant limitation of the proposed algorithm. For this reason, other strategies assessing DPD activity, such as DPD phenotyping, are investigated [4,12,13,14].

DPD converts uracil, its endogenous substrate, into dihydrouracil, and the pretreatment dihydrouracilemia (UH_2_)/uracilemia (U) ratio or uracil concentrations (U) alone have the great potential to identify patients at risk of severe fluoropyrimidine-associated toxicity [4,15]. According to certain studies, the UH_2_/U ratio correlates with 5-FU clearance and risk of toxicity; however, despite strong evidence on its clinical validity, the use of the UH_2_/U ratio in daily clinical practice is still limited [12,13,14]. In December 2018, the French drug administration made fluoropyrimidine-pretherapeutic U measurement mandatory [16]. Prescribers must clearly state on the prescription that U was considered, and the pharmacist, before delivering the drug, should ensure that the mention is written. Patients with U < 16 ng/mL have normal DPD activity; patients with 16 < U < 150 ng/mL have partial deficiency, wherein fluoropyrimidine dose should be decreased; and patients with U > 150 ng/mL have a full deficit, and thus fluoropyrimidine should be avoided.

If over-exposure is an important concern for oncologists, several papers have shown that almost 50% of patients treated with 5-FU are at risk of under-exposure, leading to sub-optimal efficacy [17,18,19,20,21,22]. 5-FU therapeutic drug monitoring (TDM) has been suggested as an option to ensure a correct exposition in all patients.

Given the evidence for DPD deficiency testing and 5-FU TDM efficacy, we conducted this retrospective study to investigate the relationship between DPD “activity” (by means of U, UH_2_/U, or *DPYD* genotyping) and 5-FU clearance in patients with gastrointestinal cancer receiving 5-FU-based regimens.

## 2. Results

### 2.1. Patient Population and Treatment

A total of 169 patients (80 females and 89 males) were included in our study. Their median age was 68 (range of 40–88) years. The main primary tumor sites were colorectal (40.8%), pancreas (31.4%), and stomach (11.2%), and were mostly metastatic (76.3%). 5-FU was administered as an intravenous bolus followed by a 46-h continuous infusion (2400 mg/m^2^ initially, then adjusted) through a portable infusion pump starting on day 1 of every cycle. Patients were treated with routinely used regimens such as FOLFIRINOX (44.4%), simplified FOLFOX-6 (26.6%), or docetaxel/FOLFOX-4 (10.6%) with or without concomitant biological therapy. Subsequent cycles were repeated every 2 weeks. 5-FU concentrations were sampled over one cycle of chemotherapy for 56 patients, two cycles for 54 patients, three cycles for 34 patients, four cycles for 17 patients, and five or more for 8 patients. Of note, some patients may have been sampled for different chemotherapy lines, but only the first one is recorded in the chemotherapy list. Median U and UH_2_/U ratio were, respectively, 10.8 [3; 37.6] ng/mL and 10.1 [3; 21.6]. Patient characteristics are listed in Table 1.

### 2.2. Intra-Individual Variability of Uracilemia and UH_2_/U Ratio

Out of the 169 patients included, 7 had two different U and UH_2_ measurements. Even if this was not intended in the analyses plan, we used those measurements to evaluate the intra-individual variability in U and UH_2_/U ratio. Mean intra-individual variabilities, 25 ± 19.7% and 26 ± 17.6% for U and UH_2_/U ratio, respectively, were not statistically different.

### 2.3. Relationships between Genotype and U or UH_2_/U Ratio

**Figure 1:**
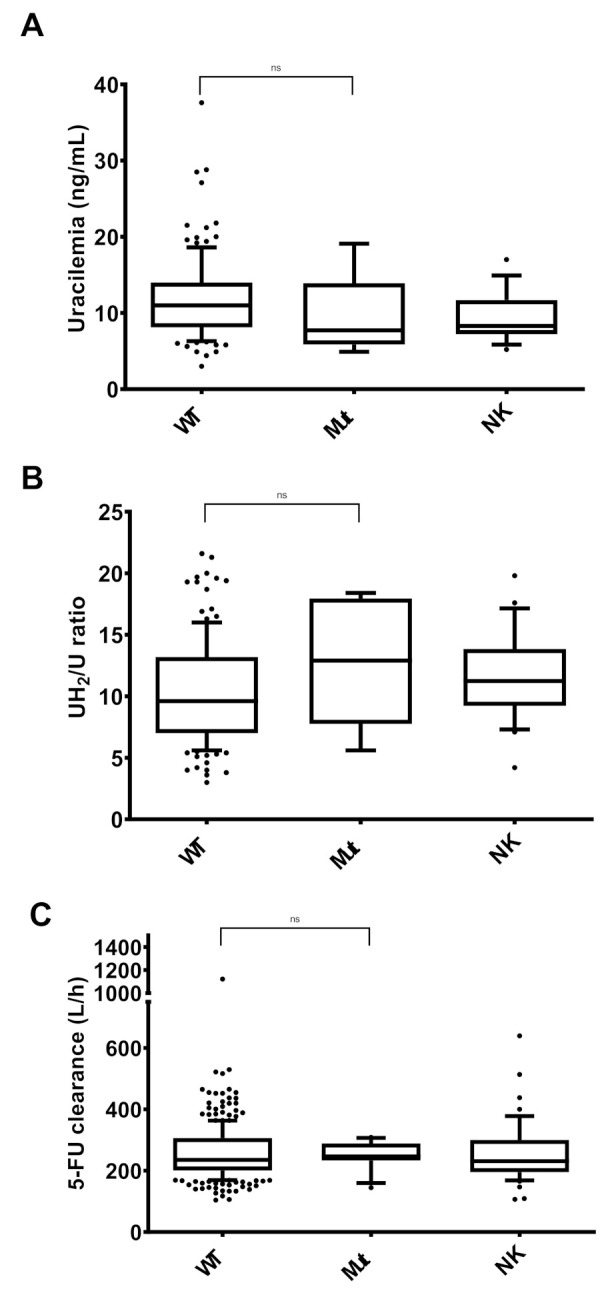
Distribution of uracilemia (**A**), UH_2_/U ratio (**B**), and 5-fluorouracil (5-FU) clearance (**C**) between patients *DPYD* wild-type (WT), heterozygous (Mut), or not known (NK). Limits of the boxplots are 10–90 percentiles. NK patients were not included in the statistical analysis. ns: non-significant.

A total of 120 genotypes were wild-type, 20 were heterozygous *6, 3 were heterozygous HapB3, 1 was heterozygous *2A, 1 was heterozygous D949V, and 24 were not known. All the heterozygous patients were grouped together in order to compare U and UH_2_/U ratio. Mean U and UH_2_/U ratio were not statistically different between both groups (mean U = 11.88 ng/mL and 9.44 ng/mL (*p*-value = 0.3138) and mean UH_2_/U ratio = 10.24 and 12.86 (*p*-value = 0.1792) in wild-type and heterozygous, respectively). This result was not different when considering wild-type and *6 patients together (Figure 1). In addition, no significant difference in terms of 5-FU clearance was observed between wild-type and heterozygous patients (*p*-value = 0.4801). Median and range are presented in Table 2.

### 2.4. Relationships between 5-FU Pharmacokinetics and U or UH_2_/U Ratio

**Figure 2:**
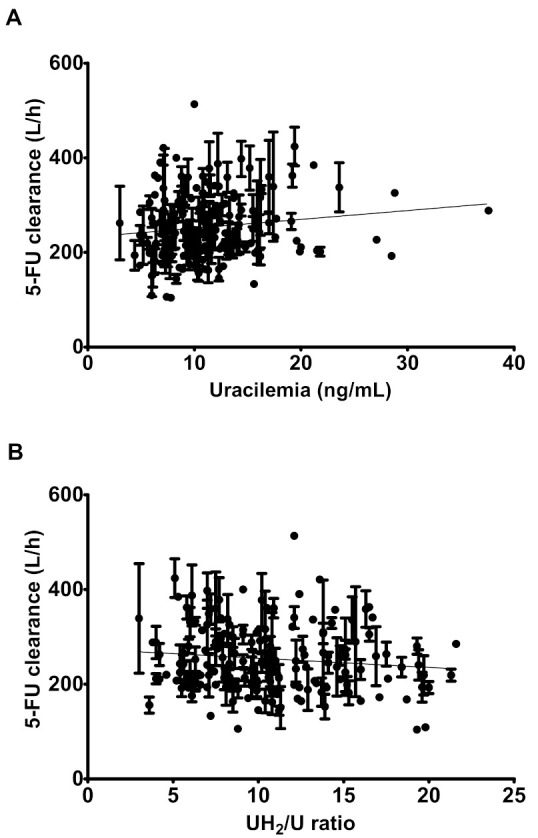
5-FU clearance versus uracilemia (**A**) or UH_2_/U ratio (**B**). Each patient calculated clearances are presented as mean and standard error to the mean. Black thin line is the regression line of the means.

No correlation was observed between 5-FU clearance and U, nor UH_2_/U ratio (Spearman’s ρ = 0.15 and −0.10, respectively), suggesting that up to 36 ng/mL (or for UH_2_/U ratio higher than 3), there was no link between 5-FU clearance and U (or UH_2_/U ratio) (Figure 2). This was confirmed by the slopes of linear regressions being not significantly different from zero (*p*-values = 0.0869 and 0.1366 for 5-FU clearance vs. U and UH_2_/U ratio, respectively).

**Figure 3:**
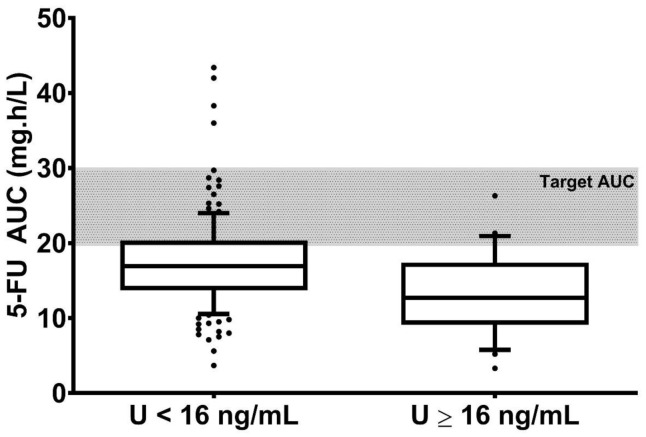
Distribution of 5-FU area under the curve (AUC) between patients with uracilemia < 16 ng/mL and ≥ 16 ng/mL. Limits of the boxplots are 10–90 percentiles.

In addition, mean 5-FU area under the curve (AUC) for patients with ≥ U16 ng/mL (13.25 mg h/L) was significantly (*p* = 0.0016) lower than for patients with U < 16 ng/mL (17.64 mg h/L) (Figure 3). However, this lower exposure may partly be explained by dose reduction in the U ≥ 16 ng/mL, as a significantly higher mean dose in the U < 16 ng/mL as compared to the U ≥ 16 ng/mL (4050 mg vs. 3540 mg, respectively; *p* < 0.001) was evidenced.

### 2.5. Dose Adaptation at Subsequent Cycles for Patients with U ≥ 16 ng/mL 

Amongst the 23 patients that had an U ≥ 16 ng/mL, 3 patients did not receive a second 5-FU infusion (due to disease progression for two patients and hematological toxicity in an UDP-Glucuronosyltransferase (UGT)1A1 heterozygous irinotecan-treated patient). None of the remaining patients had to benefit of a toxicity-related dose reduction during the first subsequent cycles. Moreover, nine (45%) had a dose increase following the TDM recommendation.

## 3. Discussion

Several strategies have been developed in order to limit the risk of toxicity after 5-FU first administration. If some centers measure directly the DPD activity in peripheral blood mononuclear cells (PBMCs) or conduct uracil breath test [23,24], the most common techniques used to evaluate DPD “activity” are by *DPYD* genotyping or by means of UH_2_/U ratio or uracilemia. For example, the French health authorities (HAS) recommend uracilemia measurement before any fluoropyrimidine treatment. The underlying hypothesis is that patients with high uracilemia have limited 5-FU clearance and thus will experience toxicities due to over-exposure. However, only limited information is available in the literature on this topic. Moreover, the best metric for DPD deficiency and boundaries to categorize a patient as deficient are still highly discussed within the scientific world [25].

Our work highlights the absence of correlation between either U or UH_2_/U ratio and *DPYD* genotype, in contrast to several other papers [4,26]. We also point out for the first time to our knowledge the absence of correlation between 5-FU clearance and *DPYD* genotype. However, these results should be taken with care, as most of our patients were wild-type and only one patient harbored a full deficient allele (*2A). Interestingly, this patient had a low U (7.7 ng/mL), but also a relatively low 5-FU clearance (mean 5-FU clearance of 188.5 L/h vs. from 240.2 to 265.7 L/h for the other genotype groups). On the other hand, the D949V patient presented a moderate U (19.1 ng/mL), but a relatively high 5-FU clearance (265.7 L/h). Our results are in contradiction with results from the largest series of pre-treatment DPD phenotyping combined with *DPYD* genotyping published by Pallet et al. [27]. This may be explained by the limited number of patients (and even more the absence of mutant homozygous patients) in our study as compared to Pallet et al., and the fact that the U variability is important within heterozygous patients. Thus, in the Pallet paper, if median U across patients harboring *2A, *13, HapB3, or D949V allele was close to our observations (between 10 and 15 ng/mL), the significant difference was highlighted because of an important number of patients.

The discrepancy between U and 5-FU clearance for those two patients brings us to the most important results of our study—the absence, in our hands, of relationships between U or UH_2_/U ratio and 5-FU clearance on the range of 3 to 37.6 ng/mL for U and 3 to 21.6 for UH_2_/U ratio. Despite a different conclusion with the pivotal study from Boisdron-Celle et al. [28], our results are not in contradiction. Indeed, in the latter case, the correlation was significant because of patients with U > 40 ng/mL that pull down the correlation. In our study, the highest U was 37.6 ng/mL; thus, we were missing those extreme patients. However, we may conclude that for U up to 30 ng/mL, there is no relationships between U and 5-FU clearance. Following the French recommendations, for patients with 16 < U < 30 ng/mL, a 5-FU dose adaptation should be triggered. However, we do believe, in light of our results, that those patients are then even at higher risk of under-exposure and that they should benefit of 5-FU TDM. This is confirmed by the fact that 45% of the U ≥ 16 ng/mL patients benefited from a dose increase between the two first cycles because of a low AUC.

Additionally, we also highlighted the intra-individual variability in U measurements. In our study, 7 patients out of 168 had two U and UH_2_ measurements. Thus, despite this limited number of patients, we calculated intra-individual variability of U and UH_2_/U ratio—both about 25%. For all enzymes, on top of pre-analytical and analytical variabilities [15,29,30,31], there is an inevitable variability due to either disposal of substrate or simply variability in the activity. Variations of endogenous uracilemia with food intake was shown by Henricks et al. [32], whereas nychthemeral variability was evidenced by Grem et al. [33]. Because of this inherent variability, some patients may have a U ≥ 16 ng/mL without being really deficient. For those patients, 5-FU TDM is the only way to ensure a correct exposure while limiting the risk of toxicity.

Out of the limited range of U in our study, the main limitation is the absence of clinical outcomes, such as toxicities. Unfortunately, the retrospective design of the study did not give us the opportunity to collect, in the patient files, consolidated data. However, information on dose modification (or interruption) between two cycles gives us a pretty good hint on the presence or not of toxicity. Indeed, oncologists follow TDM recommendations to increase dose only in case of good tolerance, and always reduce 5-FU dose (or stop it) in case of adverse events. In our study, only 13% of patients had a dose interruption (no dose reduction). Additionally, most of the patients in this group had an AUC at first cycle lower than the target AUC, which clearly underlines the under-exposure.

## 4. Materials and Methods

### 4.1. Patients 

We retrospectively reviewed the patients’ database of Dijon’s Clinical Cancer Center. All patients with gastrointestinal cancer who received a 46-h continuous 5-fluorouracil infusion-based protocol from March 2018 to January 2020 were included. All patients routinely underwent a pre-therapeutic blood analysis in order to evaluate their U and UH_2_. Additionally, 5-FU exposure was measured by means of a single blood sample during the first cycles. Of note, some patients may have received several lines of 5-FU-based chemotherapy, and 5-FU concentrations were thus measured for each line. *DPYD* genotyping was performed at the clinician discretion. All genotyped patients consented for genetic analysis, but no informed consent was required for the other patients, as samples were regular. However, data used in this manuscript were recorded in such a manner that subjects could not be identified. Patient confidentiality was maintained, and the protocol for data collection and analysis followed guidelines and was approved by our Institutional Review Board.

### 4.2. DPD Phenotyping

Uracilemia (U) and dihydrouracilemia (UH_2_) were assayed using a LC–MS/MS method. Briefly, samples were drawn, then rapidly centrifuged (within 90 min) and frozen. The extraction procedure was as follows: after addition of internal standards (isotopic U and UH_2_), ZnSO_4_ was added, followed by a mixture of CH_3_CN/MeOH (95/5), 50 µL of extemporaneous acetic acid 0.25M, and again a mixture of ethyl acetate/isopropanol (85/15). The samples were then vortexed, centrifuged, and dried under 50 °C nitrogen flow. The extracts were then reconstituted with 200 µL of H_2_O (0.1% HCOOH), and 20 µL was injected into the LC–MS device. U and UH_2_ were chromatographed using a gradient of CH_3_CN and H_2_O (both 0.1% HCOOH). This method displays lower limits of quantifications of 4.2 ng/mL and 20.0 ng/mL and is linear to 150.0 ng/mL and 650.0 ng/mL, respectively, for U and UH_2_. Using quality control (QC) samples targeted at 7.5 and 30.0 ng/mL for U and 50 and 150 ng/mL for UH_2_, the respective inter-day variabilities were of 12.3%, 9.9%, 16.0%, and 8.1% (all *n* = 22).

### 4.3. DPYD Genotyping

Genomic DNA was extracted from whole blood using a Maxwell 16 blood DNA purification kit (Promega) and Maxwell 16 instrument (Promega) according to the manufacturer’s instructions. Genomic DNA samples genotyped by Sanger sequencing for the polymorphisms of interest in this study were used as positive controls. *DPYD* polymorphisms were detected with a QuantStudio 5 instrument (Applied biosystems), using the following TaqMan Drug Metabolism SNP Genotyping Assay (Thermofisher scientific): C__11372171_10 (rs1801160, DPYD*6, V732I, c.2194C>A), C__27530948_10 (rs67376798, D949V, c.2846A>T), C__30633851_20 (rs3918290, DPYD*2A, IVS14+1G>A, c.1905+1G>A), C__11985548_10 (rs55886062, DPYD*13, I560S, c.1679T>G), C__25596099_30 (rs56038477, E412E, in haplotype B3 (HapB3), c.1236G>A).

### 4.4. 5-FU Administration, Blood Sampling, and Plasma Concentration Determination

5-FU was administered continuously through a portable pump (BodyGuard 323 Colorvison, CME, Israel), allowing a controlled flow rate over the entire 46-h. Blood samples were drawn the day following the beginning of a 46-h continuous infusion (between 8 a.m. and 10 a.m.) during the first 3 cycles. Samples were immediately centrifuged, and plasma was kept frozen at −20 °C until analyzed. Plasma 5-FU concentrations were determined by liquid chromatography. Chloro-uracil was used as internal standard. 5-FU was extracted from the plasma with isopropanol-ethyl acetate (15/85 v/v) in the presence of 200 mg ammonium sulfate to precipitate proteins. The organic phase was dried at 50 °C under nitrogen dioxide and reconstituted with 200 µL mobile phase before injection. Mobile phase consisted of methanol/water (5/95 v/v). *UV* detection was performed at 265 nm. This method was fully validated for routine measurement of 5-FU with a lower limit of quantification of 30 µg/L. The linearity was assessed from 30 µg/L to 2000 µg/L. Interday variations of the method was evaluated using two levels of QC samples (250 and 500 ng/mL). Interday precision was 10.9% and 7.9% (*n* = 9), respectively, for the 2 levels tested. Area under the curve of 5-FU concentrations vs. time (AUC) values, representing 5-FU exposure, were calculated by multiplying the 5-FU steady state concentration (mg/L) by the total infusion time (i.e., 46 h). This was made possible by the use of a portable pump, allowing a controlled flow rate. 5-FU clearance was then calculated by dividing the dose by the AUC.

### 4.5. Dose Adaptation at Subsequent Cycles for Patients with U ≥16 ng/mL

As retrospective evaluation of toxicities is not the most reliable strategy, we decided to focus on how TDM recommendations were followed. In our institution, if the dose was increased accordingly to TDM recommendations for a patient, this meant that the patient did not experience 5-FU-related toxicity. On the other hand, if the dose was not increased (or even decreased), this meant that the patient had at least one 5-FU related toxicity. Consequently, in patients with U ≥ 16 ng/mL, the fraction of patients with dose increase was calculated.

### 4.6. Data Analysis

Data are described as means ± standard deviations, median [min–max], or n (%). Intra-individual variability of U and UH_2_/U ratio was evaluated by calculating, for patients with 2 measurements, the difference, expressed as a percentage, between the highest and the lowest values. Because U and UH_2_/U ratio were not normally distributed, we used a Wilcoxon matched pairs signed rank test to compare values in patients where two measurements were performed. A *t*-test was conducted to compare U, UH_2_/U, and 5-FU clearance by *DPYD* genotype. As several 5-FU concentrations were available for most of patients, we performed regression analysis as a replicate. A linear regression was conducted to analyze the relationship between 5-FU clearance and U or UH_2_/U ratio, and slopes were compared to 0 in addition to Spearman’s correlation test in order to assess correlations between variables. Two-sided *p*-values < 0.05 were considered significant. Statistical analyses were performed using Prism 5.0 (GraphPad, CA, USA).

## 5. Conclusions

Our analysis confirms that there is a significant risk of under-exposing patients with U at least <30 ng/mL, and perhaps higher. Future studies should now focus on patients with U between 20 ng/mL and 150 ng/mL in order to characterize the correlation between U and 5-FU clearance. A focus on patients with comorbidity may also be of interest. Meanwhile, 5-FU TDM should be expanded, as it is the only way to overcome the risk of under-exposure.

## Figures and Tables

**Table 1:** Baseline patient characteristics. *N* = 169.

	Median [Min, Max]	Mean (±SD)	% (*n*)
Sex			
Male			52.7 (89)
Female			74.3 (80)
Age (years)	68 [40, 88]	67 (±10)	
Tumor localization			
Colorectal			40.8 (69)
Pancreas			31.4 (53)
Stomach			11.2 (19)
Others			16.6 (28)
Chemotherapy			
FOLFIRINOX			44.4 (75)
Simplified FOLFOX-6			28.4 (48)
Docetaxel/FOLFOX-4			10.6 (18)
Others			16.6 (28)
Biotherapy (bevacizumab, cetuximab, etc.) associated with the chemotherapy			34.9 (59)
Uracilemia (ng/mL)	10.8 [3, 37.6]	11.5 (±5.00)	
UH_2_/U ratio	10.1 [3, 21.6]	10.7 (±4.29)	
5-FU dose at first occasion (mg)	4075 [850, 5800]	3980 (±681)	
5-FU AUC at first occasion (mg)	16.6 [3.3, 42.0]	16.9 (±5.96)	
DPYD genotype			
Wild-type			71.0 (120)
*6 (heterozygous)			11.8 (20)
HapB3 (heterozygous)			1.8 (3)
*2A (heterozygous)			0.6 (1)
D949V (heterozygous)			0.6 (1)
Not known			14.2 (24)

**Table 2:** Description of U, UH_2_/U, and 5-FU clearance distribution based on DPYD genotype.

	Uracilemia (ng/mL) Median [Min, Max]	UH_2_/U Ratio Median [Min, Max]	5-FU Clearance Median [Min, Max]
*DPYD* wild-type	11 [3, 37.6]	9.6 [3, 21.6]	235.0 [104.2, 1121]
*DPYD* mutated	7.7 [4.9, 19.10]	12.9 [5.6, 18.4]	246.4 [144.6, 308.5]
